# Immunogenicity of Three Different Influenza Vaccines against Homologous and Heterologous Strains in Nursing Home Elderly Residents

**DOI:** 10.1155/2010/517198

**Published:** 2010-03-29

**Authors:** Vincenzo Baldo, Tatjana Baldovin, Michele Pellegrini, Gabriele Angiolelli, Silvia Majori, Annarosa Floreani, Marta Cecilia Busana, Chiara Bertoncello, Renzo Trivello

**Affiliations:** ^1^Department of Environmental Medicine and Public Health, Institute of Hygiene, University of Padua, Via loredan 18, 35151 Padua, Italy; ^2^Global Clinical Research and Development, Novartis Vaccines & Diagnostics S.r.l., Via Fiorentina 1, 53100 Siena, Italy; ^3^Local Health Unit n.13, Veneto Region, Via L. Mariutto, 76-30035 Mirano, Italy; ^4^Department of Medicine and Public Health, Section of Hygiene and Preventive, Environmental and Occupational Medicine, University of Verona, Strada Le Grazie, 8-37134 Verona, Italy; ^5^Department of Surgical and Gastroenterological Sciences, University of Padua, Via Giustiniani 2, 35100 Padua, Italy

## Abstract

We studied whether MF59-adjuvanted influenza vaccine improves immunity against drifted influenza strains in institutionalised elderly with underling chronic health conditions. Sera from a randomized study, comparing MF59-adjuvanted (Sub/MF59, *n* = 72), virosomal (SVV, *n* = 39), and split (*n* = 88) vaccines, were retested using a hemagglutination inhibition (HI) assay against homologous (Northern Hemisphere [NH] 1998/99) and drifted (NH 2006/07) strains. Corrected postvaccination HI antibody titres were significantly higher with Sub/MF59 than SVV for all strains; GMTs against homologous A/H3N2 and B and both drifted A strains were significantly higher for Sub/MF59 than split. Seroprotection rates and mean-fold titer increases were generally higher with Sub/MF59 for all A influenza strains. MF59-adjuvanted influenza vaccine induced greater and broader immune responses in elderly people with chronic conditions, than conventional virosomal and split vaccines, particularly for A/H1 and A/H3 strains, potentially giving clinical benefit in seasons where antigenic mismatch occurs.

## 1. Introduction

The frequency and severity of infectious diseases, including influenza, increase with old age. The elderly are particularly vulnerable to influenza and this highly contagious infectious disease causes a high frequency of morbidity and mortality in older individuals [1–4]. The mortality rate in the elderly is particularly high compared with the general population, with 95% of all influenza-related deaths occurring in the elderly, primarily in those with underlying chronic health conditions [5]. 

Groups at high-risk of complications of influenza include patients with cardiovascular or pulmonary conditions, metabolic diseases, and the institutionalized [6]. In fact, influenza can exacerbate underlying diseases in the elderly population, being the likely primary cause of the winter-season increase in mortality in patients with ischemic heart disease, cerebrovascular disease and diabetes mellitus [7, 8]. 

Annual vaccination is the recommended method to prevent influenza; the WHO has suggested that vaccination can reduce influenza-related morbidity by 60% and influenza-related mortality by 70–80% [9]. However, currently available influenza vaccines have demonstrated limited efficacy in the elderly, mainly because of the waning immune response typical with advancing age [10–12]. Indeed, lower IgA and IgG antibody responses, delayed peak antibody titers, and a faster decline in titers following vaccination are observed, especially in very old and frail persons [13].

The continual evolution of the influenza virus also impacts on the effectiveness of influenza vaccines. Antigenic drift frequently occurs in influenza A and B subtypes and the impact of this drift on vaccine effectiveness in the elderly is considered very high [14–16]. It has been suggested that antigenic drift is associated with a more severe and early onset of influenza epidemic, since the level of preexisting immunity to the drifted strain is reduced [17]. In elderly subjects seroprotection rates can be as low as 20% against drifted strains, dropping from ≥70% in years where a good antigenic match is observed [18–21].

Meeting the challenge presented by waning immunity in the elderly requires vaccines that offer enhanced immunogenicity and increased clinical protection, such as adjuvanted influenza vaccines. Formulation of a subunit influenza vaccine with the MF59 adjuvant has been shown to enhance immunogenicity and offer broader serological protection in the elderly compared with conventional non-adjuvanted vaccines, especially versus the most epidemiologically prevalent A/H3N2 influenza viruses [6, 20, 22].

This study was performed to assess the immunogenicity of three inactivated influenza vaccines, a MF59-adjuvanted subunit vaccine (Sub/MF59; FLUAD^®^, Novartis Vaccines), a virosomal vaccine (SVV; InflexalV^®^, Swiss Serum and Vaccine Institute), and a split vaccine (Split; Mutagrip^®^, Pasteur Merieux MSD), against homologous and heterologous strains, by retesting sera of elderly nursing home residents with chronic underlying conditions, who participated in a previous randomized, controlled trial [6].

## 2. Materials and Methods

Sera from a subset of 199 elderly nursing home residents (≥65 years of age) previously enrolled in a randomized, controlled trial performed during the winter season of 1998/99 [6], were reanalyzed to test the immunogenicity conferred by MF59-adjuvanted influenza vaccine (Sub/MF59; *n* = 72), by a virosomal (SVV, *n* = 39) and a split (Split; *n* = 88) vaccines against homologous and heterologous influenza strains.

During the clinical study, after obtaining informed consent, blood samples (approximately 10 mL) were drawn prior to and 4 weeks after vaccination. Sera were stored at −20°C until laboratory determination of HI antibody titres, as described elsewhere [23].

All subjects received a single 0.5 mL intramuscular (IM) dose of Sub/MF59, virosomal or split vaccine in the deltoid region of the non-dominant arm. Each vaccine dose contained 15 *μ*g of hemagglutinin of each of the influenza strains recommended by the WHO for the 1998/99 Northern Hemisphere (NH) influenza season: A/Sydney/5/97(A/H3N2)-like virus, A/Beijing/262/95(A/H1N1)-like virus and B/Beijing/184/93-like virus.

The study was conducted in compliance with the Italian Law Decree on the protection of personal data.

### 2.1. Assessment of Vaccine Immunogenicity

The current analysis tested the immunogenicity using the HI assay against the homologous 1998/99 strains and the heterologous viral strains recommended for the 2006/07 NH influenza season [A/Wisconsin/67/2005 (A/H3N2)-like virus; A/New Caledonia/20/99 (A/H1N1)-like virus and B/Malaysia/2506/2004-like virus]. The heterologous B strain selected for immunogenicity analyses belongs to the B/Victoria/2/87 lineage, whereas the B strain included in the vaccine formulation (B/Beijing) was from the alternative phylogenetic line of B/Yamagata/16/88, with a previously reported lack of cross-reactivity between antibodies for these two divergent lineages [24]. 

**Figure 1:**
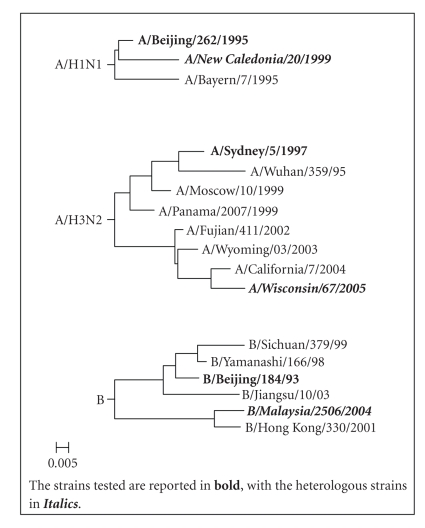
Hemagglutinin phylogenetic trees of the strains recommended for NH vaccine formulation from 1997/1998 to 2006/2007 influenza seasons.

Phylogenetic trees of the influenza strains analyzed in the study (both homologous and heterologous), including the vaccine strains recommended for NH formulation from 1997/98 to 2006/07 influenza seasons, was based on sequence analysis of the region codifying for the hemagglutinin (Figure 1). Data on influenza strains was obtained from the website of the National Institute of Allergy and Infectious Diseases-funded Influenza Research Database (NIAID IRD; http://www.fludb.org), and phylogenetic analyses were conducted using the software Molecular Evolutionary Genetics Analysis (MEGA), version 4.0 [25].

The study parameters considered as expression of humoral immune response were: the Geometric Mean Titers (GMTs) of HI antibodies with 95% confidence intervals (CI); post-vaccination mean-fold increase (MFI) in HI antibodies (ratio of post- to pre-vaccination titres); the number of subjects with protective HI antibody level (titre ≥ 40 was considered protective); and the number of subjects with at least a 4-fold increase in post-vaccination titres.

### 2.2. Statistical Analysis

Data were analyzed using the Statistical Package for the Social Sciences (SPSS, Chicago, IL, USA). The chi-square test was performed to analyze differences between proportions of subjects. Statistical significance between pre- and postvaccination titres was calculated using the paired Student's *t*-test. Comparison of different vaccine groups was determined by Student's *t*-test for unpaired data. 

A multivariate logistic regression analysis was carried out for each virus strain to determine variables that are independently associated with the likelihood of a person achieving at least a 4-fold increase in HI titres. A *P*-value of <.05 was considered to indicate statistical significance, with an odds ratio (OR) of <1 signifying that a person was less likely to achieve a 4-fold increase.

Correction of the GMTs for pre-vaccination status was performed: absolute titres were divided by 5 (i.e., undetectable titre <10) and then transformed to binary logarithms. On this scale, an undetectable (seronegative) titre represents “0”, a titre of 10 represents “1”, a titre of 20 represents “2”, and so forth, HI titres were transformed into binary logarithms and corrected for pre-vaccination status as described by Beyer et al. [26].

## 3. Results

Sera were available from 199 elderly who took part into the original study, and were retested for these new immunogenicity analyses for the Sub/MF59 (*n* = 72), SVV (*n* = 39) and Split (*n* = 88) groups. According to the original baseline characteristics more healthy subjects populated the split group, compared with Sub/MF59 and SVV. The majority of subjects in these last two groups (87.5% and 79.5%, resp.) had at least one underlying chronic condition, including cardiac and pulmonary conditions, or diabetes mellitus. More than 80% of subjects in each group were >75 years of age. The demographic characteristics of the subjects, recorded at time of the original study, are summarized in Table 1. 

No statistically significant differences were found in terms of baseline GMTs between vaccine groups versus homologous or heterologous strains. 

**Figure 2:**
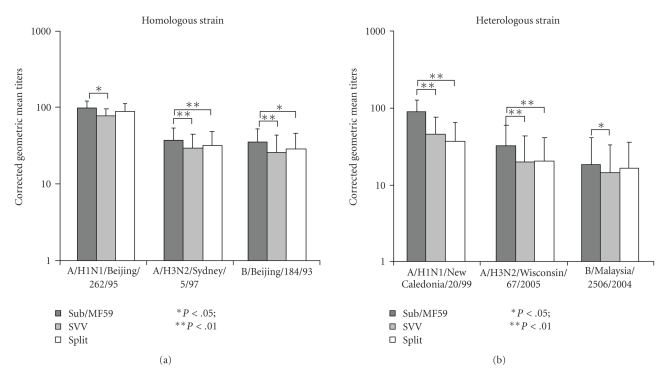
GMTs against homologous and heterologous influenza strains, according to vaccine group.

The antibody responses are shown in Table 2 and Figure 2, according to viral strain and vaccine group. Vaccination elicited higher GMTs against all strains, in all three vaccination groups (Figure 2). 

### 3.1. Immunogenicity against Homologous Strains

**Figure 3:**
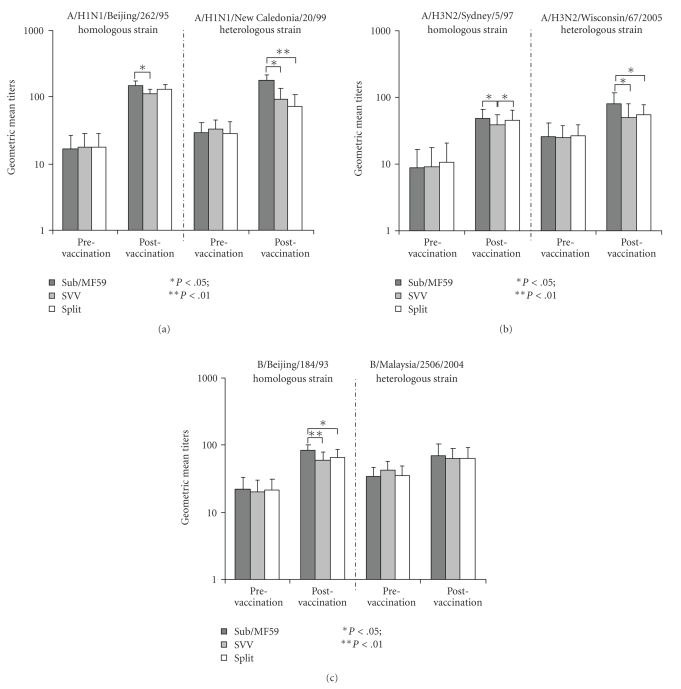
Corrected GMTs against homologous and heterologous influenza strains, according to vaccine group.

Very high seroprotection rates (SP) were achieved by all three vaccines (Table 2), but vaccination with Sub/MF59 resulted in slightly but consistently higher postvaccination GMTs than the other two vaccination groups (Figure 2). In particular, Sub/MF59 induced statistically significantly higher HI antibody titers for all three strains, than those of the SVV vaccine group. The GMTs were substantially confirmed after correction for baseline titers (Figure 3), and Sub/MF59 vaccine showed the ability to induce statistically significant (*P*  <  .01) higher GMTs against A/H3 and B homologous strains, compared to the split vaccine.

Postvaccination mean-fold increases in HI antibodies were greater with Sub/MF59 compared with both virosomal and split vaccines (Table 2).

Significantly more subjects achieved at least a 4-fold increase in HI titres against the homologous B strain following Sub/MF59 vaccination, than the virosomal vaccine (66.7% versus 46.2%, resp., *P* =  .03) (Table 2).

### 3.2. Immunogenicity against Heterologous Strains

Consistently higher GMTs were reported in the Sub/MF59 group, and these values were statistically significant for the A/H3N2 and A/H1N1 strains (Figure 2), compared with both non-adjuvanted vaccines. After Beyer's correction, the HI antibody titers against B strains were significantly higher in the Sub/MF59 than in the virosomal group.

Post-vaccination mean-fold increases in HI antibodies were greater with Sub/MF59 compared with the other two vaccine groups against all the three influenza virus variants (Table 2).

Seroprotection rates induced by Sub/MF59 group were higher than those after the SVV injection for both A strains (*P*  <  .01 versus H1N1, *P*  <  .05 versus A/H3N2). Similar SP rates against the B drifted strain were found in the three vaccine groups.

Significantly more subjects achieved at least a 4-fold increase in titres following Sub/MF59 vaccination against all three heterologous strains compared with SVV, and against both A drifted strains when compared with split group (Table 2). The rate of 4-fold increase in HI titers versus B heterologous strain was significantly higher in the split group compared with SVV, and similar to the Sub/MF59 group. 

As shown in Table 3, the multivariate analysis of variables associated with 4-fold increase in HI antibody titers revealed that the presence of previous vaccination, only for the B strain, and the type of vaccine used, for all strain, were strong predictors of immune response against heterologous influenza strains.

## 4. Discussion

Vaccination is of crucial importance in preventing infection and protecting the vulnerable elderly population from disease but, over the past decade, a large number of studies have shown that antibody responses after vaccination are lower in elderly persons than in young adults, and that a variety of vaccines are less efficient in elderly persons [27]. 

Although the relationship between specific anti-influenza virus antibody levels and clinical protection is intrinsically variable [28], with other factors contributing to protection, such as antibodies to neuraminidase [29] and cellular immunity [30], antibody titers against hemaglutinin and derived surrogate end-points are currently considered the basis for licensure of influenza vaccines in the different age groups.

The use of an adjuvant to enhance the antibody response has been considered for a long time to be a valid option to increase the efficacy of influenza vaccines [31, 32]. Preclinical studies have shown that the adjuvant MF59 is internalized by dendritic cells at the site of injection and facilitates internalization of the antigen to enhance the immune response and produce more antibodies. 

This study was performed to evaluate the immunogenicity, using a validated HI assay, of three different inactivated influenza vaccines against both homologous and heterologous influenza strains in all evaluable sera from an original trial in nursing elderly residents with chronic underlying diseases. The original study population mainly comprised very elderly individuals, most of them with at least one underlying medical condition.

Although the sera were retested approximately 10 years after the original study, the data clearly confirmed the previous published evaluations vs. homologous antigens, showing higher immunogenicity in the adjuvanted vaccine group and the lower immune responses in the virosomal group [6]. After correction according to pre-vaccination status, a statistically significant difference in HI titers was evident in favor of MF59-adjuvanted vaccine against all three vaccine strains, when compared with the split vaccine, and against A/H3N2 and B antigens when compared with the split vaccine. Although all three inactivated vaccines induced very high seroprotection rates, 4-fold increase in titers was consistently higher in the MF59-adjuvanted vaccine group.

In addition, the MF59-adjuvanted influenza vaccine induced a higher level of immunogenicity against strains that bear no close antigenic similarity to those included in the vaccine formulation. For both heterologous A influenza strains, Sub/MF59 induced significantly higher HI antibodies and 4-fold increases in titers than the two non-adjuvanted comparators. For the B drifted strain, significantly higher HI titers were induced by MF59 adjuvanted vaccine, compared with SVV, and both adjuvanted and conventional split vaccine showed the highest increases in titers, compared with the virosomal vaccine.

Multivariate analysis also revealed that the presence of previous vaccination and the type of vaccine used were strong predictors of immune response against heterologous influenza strains (Table 3). 

All vaccines fulfilled all three CHMP criteria for elderly against homologous strains. Against A heterologous antigens, only Sub/MF59 fulfilled all CHMP criteria versus A/H3N2 and A/H1N1 strains, SVV met all criteria against A/H1N1 and none of them for A/H3N2, Split vaccine fulfilled only one criterion against both antigens. Against B drifted strain only one criterion was met (seroprotection rate) by all vaccines tested.

In conclusion, data from these immunogenicity analyses confirmed that MF59-adjuvanted influenza vaccine can offer greater and broader immunogenicity against influenza in elderly and very elderly individuals who are at high risk of influenza-related complications. 

Studies to further evaluate the relationship between vaccines-induced immunogenicity against homologous and heterologous strains and clinical protection are clearly warranted.

## Figures and Tables

**Table 1:** Baseline characteristics of the study groups.

Subgroup	Sub/MF59 (*n* = 72)	Split (*n* = 88)	SVV (*n* = 39)
	*n* (%)	*n* (%)	*n* (%)
Age group, years			
≤85	31 (43.1)	42 (47.7)	11 (28.2)
>85	41 (56.9)	46 (52.3)	28 (71.8)
Gender			
Males	5 (6.9)	24 (27.3)	0 (0.0)
Females	67 (93.1)	64 (72.7)	39 (100)
Previously vaccinated			
No	12 (16.7)	16 (18.2)	2 (5.1)
Yes	60 (83.3)	76 (81.8)	37 (94.9)
Underlying disease			
No	9 (12.5)	35 (39.8)	8 (20.5)
Yes*	63 (87.5)	53 (60.2)	31 (79.5)
Heart condition	51 (70.8)	49 (55.7)	24 (61.5)
Lung condition	42 (58.3)	11 (12.5)	17 (43.6)
Diabetes mellitus	12 (16.7)	8 (9.1)	3 (7.7)
Other	14 (19.4)	2 (2.3)	9 (23.1)

*More than one risk status was possible for each subject.

**Table 2:** Mean Fold Increase in titres (MFI), Seroprotection (≥40), and 4-fold increase rates according to viral strain and vaccine group.

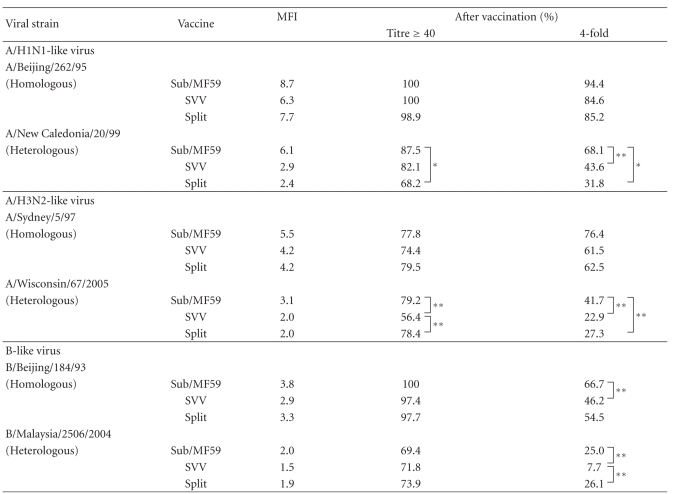

*P  <  .01; **P  <  .05.

**Table 3:** Multivariate analysis of variables associated with 4-fold HI-antibody titer increases according to heterologous virus strains.

Variables	A/H1N1/NewCaledonia/20/99		A/H3N2/Wisconsin/67/2005		B/Malaysia/2506/2004	
	OR	95% CI	OR	95% CI	OR	95% CI
Age (<85)	0.784	(0.421–1.458)	1.515	(0.829–2.768)	0.681	(0.344–1.347)
Gender (males)	0.465	(0.177–1.219)	1.280	(0.507–3.234)	1.239	(0.463–3.317)
Underlying diseases	1.431	(0.783–2.615)	0.897	(0.490–1.644)	1.598	(0.811–3.149)
Previous vaccinations	1.447	(0.738–2.835)	0.806	(0.417–1.559)	0.307	(0.151–0.626)
Prevaccination titre (≥1 : 40)	1.803	(0.976–3.333)	0.676	(0.355–1.286)	0.891	(0.447–1.776)
Split versus Sub/MF59	0.287	(0.150–0.547)	0.436	(0.226–0.842)	0.795	(0.404–1.564)
SVV versus Sub/MF59	0.400	(0.180–0.893)	0.402	(0.167–0.965)	0.215	(0.060–0.774)
